# Supplementary material for: Effect of the First Feeding on Enterocytes of Newborn Rats
Source: Int J Mol Sci. 2022 Nov 16;23(22):14179. doi: 10.3390/ijms232214179 (PMC9699143; doi:10.3390/ijms232214179)
Supplement: Supplementary file 1 [file ijms-23-14179-s001.zip › ijms-1945412-supplementary-for xml.pdf]

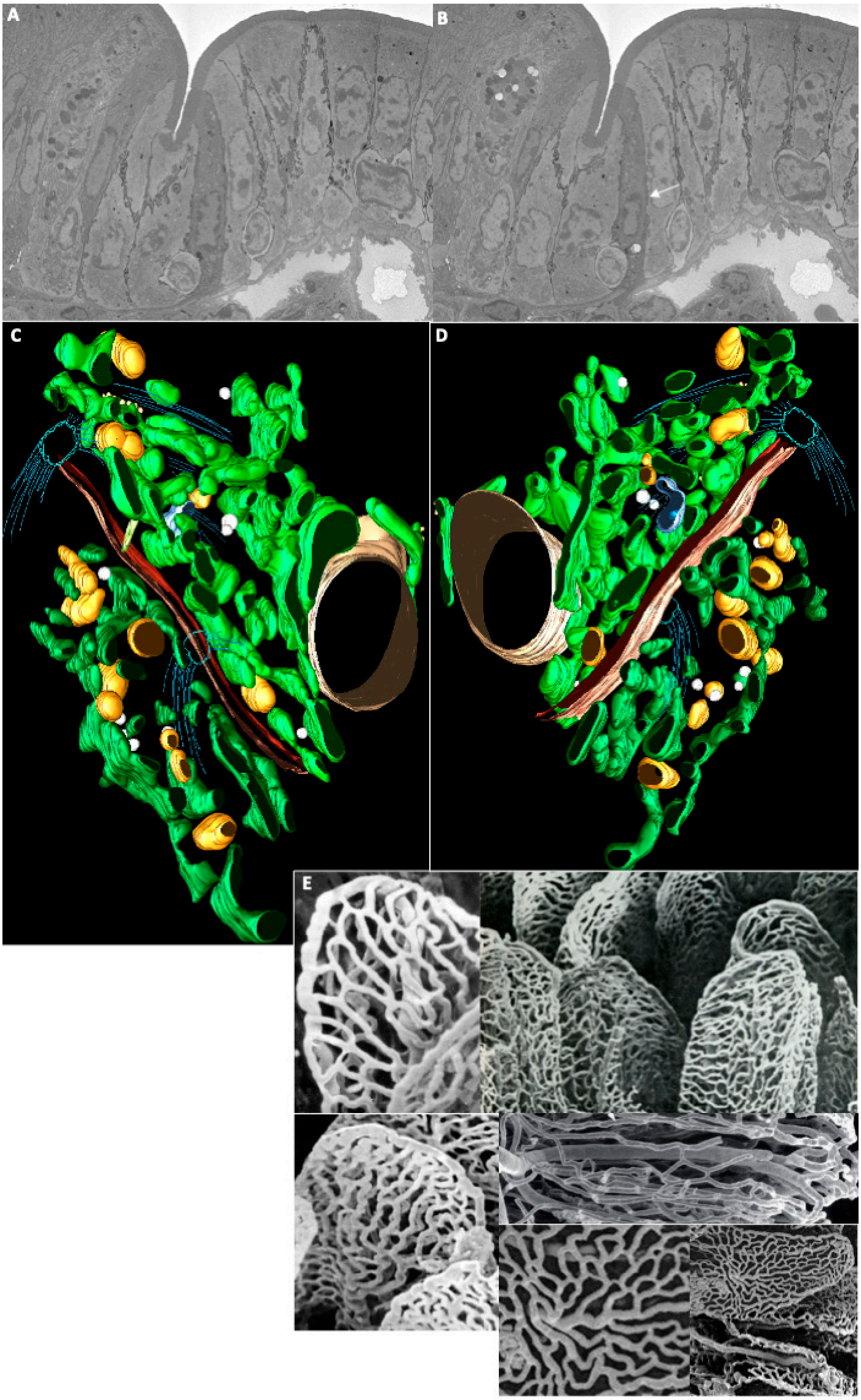
A
B
C
D
E

Figure S1. Structure of enterocytes and blood micro-vessels in adult rats. (A, B) General view of enterocytes in the adult rat. Serial images obtained with 3VIEW. (C,D) Rotation of the 3D model of the tubular networks formed by the smooth ER near the BLPM (brown planes.) Yellow are endosomes; green is the smooth ER. Light brownish is multivesicular body, which forms contacts sites with the BLPM. (A, B) Serial images of enterocytes obtained with 3VIEW. (C, D) 3D model 0f the tubular network situated near the BLPM of enterocyte. (E) Corrosion casts of the blood micro-vessels of intestine villi (from [4]). Scale bars see in [4].
